# Interventional strategies for the management of the disruptive physician: A systematic review

**DOI:** 10.1016/j.fhj.2025.100448

**Published:** 2025-06-30

**Authors:** Allen M. Chen

**Affiliations:** Department of Radiation Oncology, University of California, Irvine, Chao Family Comprehensive Cancer Center, Orange, CA 92868, USA

**Keywords:** Professionalism, Leadership, Management, Workforce, Disruptive

## Abstract

**Purpose:**

The aim of this study was to identify and evaluate published interventional strategies that address disruptive physician behaviour.

**Methods and materials:**

This study was designed based on the Preferred Reporting Items for Systematic Review and Meta-Analysis Protocols (PRISMA-P) statement. A comprehensive literature search of publications from January 2013 to November 2023 was undertaken to identify original peer-reviewed studies pertaining to the development and implementation of targeted strategies for the management of disruptive physicians. An interpretive synthesis of the available publications was then presented in both tabular and descriptive format.

**Results:**

Among the 1,005 original articles that entered the final selection process, a total of 998 were then excluded because they were focused solely on the characterisation of disruptive behaviour (n=399); limited to trainees (n=258), limited to ancillary staff (n=135); concentrated on prevention (n=111); and described consequences (n=95). A total of seven original publications thus met criteria for inclusion and differed in their design, methods and endpoints. Two articles reported on longitudinal experiences involving prospective data. The most commonly identified themes related to accountability (five studies), organisational commitment (four studies) and professionalism training (three studies), with most studies covering more than one item.

**Conclusions:**

Given the practical scope of this problem, a relative lack of publications exist focused on remediation strategies for disruptive physician behaviour. Investment in the development of evidence-based programmes are warranted.

## Highlights

• The prevalence of incivility is increasing in the healthcare workplace.
• A surprisingly limited number of studies exist on interventional strategies to address a disruptive physician.
• The need for future research in this area is highlighted.

## Introduction

The American Medical Association (AMA) has defined disruptive behaviour as ‘personal conduct – verbal or physical – that has the potential to negatively affect patient care or the ability to work with other members of the healthcare team’.^1^ While statistics have estimated that approximately 3–5% of all physicians can be categorised as disruptive, the prevalence of misbehaviour seems to be more pervasive.^2^ This is because disruptive conduct can be a one-time, isolated event or, more ominously, representative of a larger pattern. In a classic survey of nurses and physicians at >100 hospitals, a staggering 77% of respondents reported witnessing physicians engage in disruptive behaviour, which was most typically classified as verbal abuse of another employee.^3^

Given the potential of disruptive physicians to compromise patient care and/or workplace morale, the need to evaluate interventions for remedial purposes is of practical relevance. Indeed, the Joint Commission expects hospitals to develop codes of conduct, define behaviour norms, and detail consequences for deviation from those norms, and ‘establish a process for managing disruptive behaviors’.^4^ However, many healthcare leaders are not sufficiently trained to manage disruptive physicians. Furthermore, the lack of institutional support and/or resources can make the enforcement of consequences for perpetrators challenging. In a survey of the membership from the American College of Physician Executives, only 17% of male and 11% of female physician executives strongly agreed that they were ‘well prepared to deal with disruptive behaviour’, and 61% of respondents desired more training in confronting disruptive behaviour.^5^

Although numerous studies have been published detailing the scope and scale of disruptive behaviour in healthcare, relatively few articles have focused on strategies for remediation.^6^, ^7^, ^8^, ^9^, ^10^, ^11^, ^12^, ^13^, ^14^, ^15^, ^16^, ^17^, ^18^ Despite the agreement that disruptive behaviour is increasingly problematic, questions persist on how to manage offenders. Indeed, one survey of hospital staff showed that only 13% of respondents felt that healthcare organisations had good enforcement procedures to handle disruptive behaviour.^19^ To address these needs, a systematic review was undertaken with the goal of identifying and evaluating strategies for the management of disruptive physician behaviour.

## Methods and materials

The design of this review originated from an internal grant that was awarded for the purpose of developing educational tools to raise awareness of professionalism issues, specifically as they relate to inclusion, civility and conduct in the setting of an expanding workforce. This systematic review was carried out following the Preferred Reporting Items for Systematic Reviews and Meta-Analyses (PRISMA) guidelines with the objective of identifying peer-reviewed publications reporting on strategies for the management of disruptive physicians. The initial screen was conducted on 30 October 2023, and repeated again on 30 November 2023, 12 January 2024 and 14 February 2024.

To start, a MEDLINE literature search of publicly accessible publications was undertaken to identify original peer-reviewed works pertaining to the topic of interventional strategies to address incivility in the healthcare workplace. The search terms ‘professionalism’, ‘civility’, ‘conduct’, ‘empathy’, ‘humanism’, ‘inclusion’, ‘bullying’, ‘teamwork’, ‘conflict’, ‘disruptive’, ‘remediation’ and ‘communication’ were broadly inputted in various permutations through the MEDLINE database to comprehensively initiate this exercise. To ensure that all possible publications were captured, multiple iterations of the search was processed. Boolean operators were routinely used to combine search terms, and advanced field tags were incorporated to refine the selection process in an attempt to limit the analysis to clinically oriented papers focused on healthcare. Reference lists from included articles were cross-checked to identify additional articles. Review articles, narratives, editorials, commentaries, and papers presented as conference proceedings were excluded, as well as those originating from areas outside the healthcare domain. Articles published from January 2013 to November 2023 with full text available and restricted to the English language and human subjects were included. The full bibliographies of identified articles were reviewed; and irrelevant studies and/or those of insufficient quality were selectively removed at the discretion of the investigator. Where individual works were included in multiple published series, the most complete or recent article was cited. To measure quality of the studies the National Institutes of Health’s quality-measure tool for optimising internal validity was used, and only those studies rated as ‘good’ or ‘fair’ were included.^20^ An interpretive synthesis of the available publications was then presented in both tabular and descriptive format.

## Results

The initial search yielded 10,755 independent articles. After broad screening of these publications based on title and abstract, a total of 2,776 studies proceeded to full-text screening and were downloaded for review. Subsequently, 1,771 articles were excluded because they were irrelevant to the topic of disruptive behaviour (N=902); review articles (N=371); opinion pieces, letters or editorials (N=365); narratives (N=71); conference proceedings or position papers (N=39); or duplicative from prior works (N=14). Another nine publications were excluded because they originated from non-medically related fields and/or were designed from a perspective outside mainstream medicine.

Among the 1,005 original peer-reviewed articles that entered the final stage of the selection process and were reviewed in depth, a total of 998 were then excluded for the following reasons: focused on the characterisation of disruptive behaviour with respect to prevalence, magnitude and/or severity without proposing any interventional strategies (n=399); focused on disruptive behaviour in medical students and/or physicians-in-training (n=258); focused on disruptive behaviour among nurses, medical assistants, and/or ancillary staff exclusively (n=135); focused more on the identification of risk factors and/or determinants for disruptive behaviour with more preventive than interventional intent (n=111); focused on the consequences of disruptive behaviour (n=95).

**Fig. 1:**
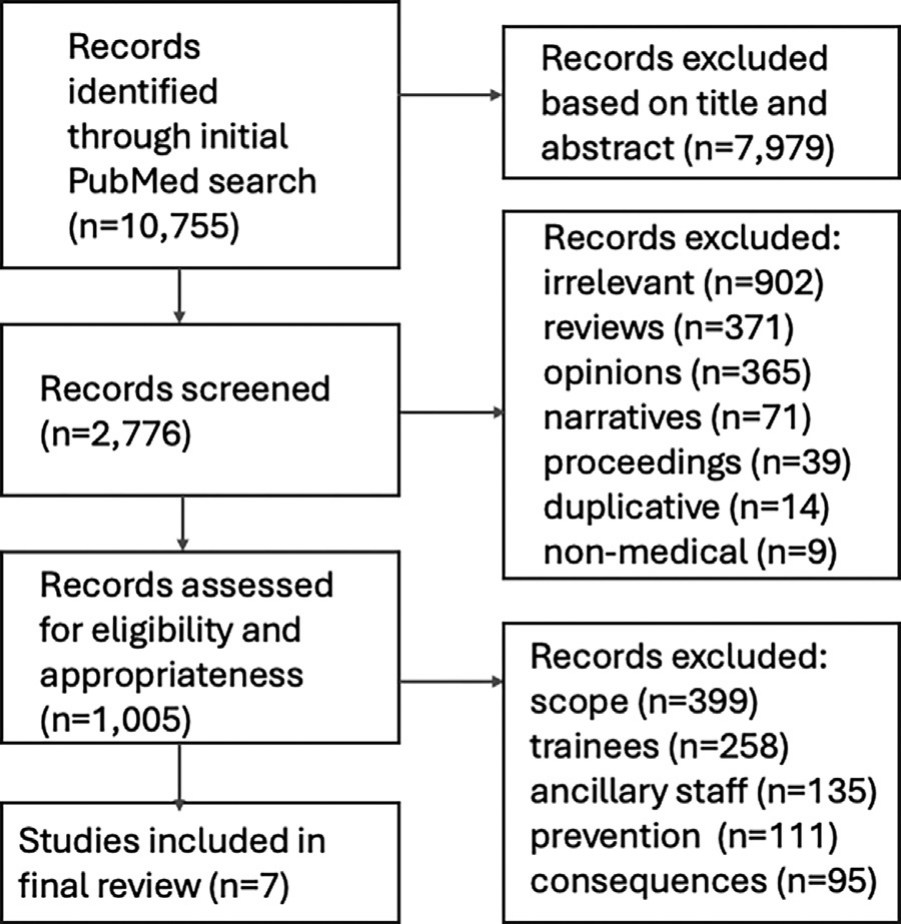
Schematic illustration of the search strategy.

A total of seven original peer-reviewed publications thus met eligibility criteria and formed the basis for this systematic review. A schematic illustration of the flowchart outlining the results of the search strategy is shown in Fig. 1. All seven publications identified were single-institutional reports. Five publications originated from academic medical centres; one was from a medical centre affiliated with the armed forces; and one was from private industry. Six were written by investigators in the USA and one was from Singapore. Two studies reported on longitudinal experiences involving prospective data collection. The most commonly identified themes were setting expectations (five studies), organisational commitment (four studies) and professionalism training (three studies). Most articles covered multiple themes. Table 1 outlines the details of the selected studies and provides a descriptive summary of key findings from the seven peer-reviewed publications that were identified.

**Table 1:** Descriptive summary of identified publications.

Authors	Year	Intervention	Key findings	Reference
Webb *et al*^24^	2016	CORS	Qualitative and quantitative data obtained over a 3-year period involving 372 reports of disruptive behaviour showed that co-worker intervention and organisational guidelines limited the number of repeat offenders. Since the inception of CORS, 71% of those individuals who received a report of concern were not named in any subsequent reports during 1-year follow-up period after the behaviour was brought to their attention.	24
Swiggart *et al*^25^	2020	PDP	A total of 24 of 28 physicians who completed PDP (3 days followed by 3 day-long sessions spread over 6 months) which focused on systematic education in emotional intelligence, self-awareness, introspection, interpersonal skills, conflict resolution, leadership, self-care, emotional regulation and mindfulness improved future behaviour based on pre- and post-course testing. Positive behaviours that increased after PDP included teamwork, peer relations, family balance and empathy.	25
Williams and Williams^26^	2020	EVLA	The EVLA framework attempts to address the underlying contributing factors and the environment in which the behaviour occurs. The five components: Capacity, Capability, Readiness, Action and Continuity. Interventions include assessment of health aspects that may contribute to misconduct and also the cognitive/emotional beliefs and values that provide the basis of behaviour.	26
Finlayson *et al*^29^	2013	FFDE	Data from 381 physicians referred for remediation showed that FFDE was effective at identifying physicians who might be unsafe to practise. Notably, physicians evaluated for disruptive behaviour were found to be at significantly higher risk of being unfit than those referred for substance abuse, mental health and/or sexual misconduct. Evaluating physicians referred for FFDE determines whether those referred for disruptive behaviour are more or less likely to be declared fit for duty than those referred for mental health, substance abuse or sexual misconduct.	29
Hastie *et al*^27^	2020	3E model	3E model that focuses on cultural change through establishment of norms, education and empowerment. What is emphasised is a collective effort with input from all organisational leaders. The norms of conduct are built by an organisation, enforced by its leaders, and upheld by its members. Our learning and working environments should reflect those standards of professionalism, where deviations from behaviour norms are not tolerated. Individuals should feel empowered to speak up and to come forward, whether they are targets or bystanders of disruptive behaviour, to protect their patients, their colleagues and themselves.	27
Junga *et al*^28^	2019	DEAL	Four-step ‘DEAL’ model focused on determining the stakes, explaining intent, assessing consequences, and leveraging the future. This presents a general framework for on how to approach negotiations based on the four archetypes of the disruptive physician: the know-it-all, the insecure, the flake and the combatant.	28
Lim *et al*^19^	2021	Three-prong Approach	Based on questionnaires sent to 1,218 physicians and nurses, of which 500 replied, the authors developed a qualitative framework for addressing disruptive behaviour based on respondent priorities. The three-prong approach focuses on 1) deterrence (feedback systems, reporting mechanisms), 2) development (training in communication, self-awareness, emotional intelligence), and 3) demonstration (organisational leadership).	

Abbreviations: PDP, programme for distressed physicians; CORS, co-worker observation reporting system; EVLA, environmentally valid learning approach; FFDE, fitness for duty evaluation; DEAL, determine the stakes, explain the intent, assess the consequences, leverage the future.

## Discussion

The most striking finding from this study relates to the relative paucity of publications identified focused on remediation strategies for disruptive behaviour among physicians. This is surprising given the scope of this problem and its associated threats to patient safety, team collaboration and workforce cohesiveness.^21^, ^22^, ^23^ Indeed, while many publications have focused on the identification and characterisation of disruptive conduct, few have offered tangible solutions.^6^, ^7^, ^8^, ^9^, ^10^ While this possibly speaks to the complexities and challenges of managing disruptive physicians, it does not diminish the importance of developing practical and sustainable approaches for intervention.

Notably, the seven studies that were identified in this systematic review varied significantly in size, design and perspective.^19^^,^^24^, ^25^, ^26^, ^27^, ^28^, ^29^ Most were small, retrospective experiences that were largely anecdotal and descriptive in nature, and all originated from a single institution. Nonetheless, some notable themes emerged across these studies that might be useful to develop a general framework for addressing disruptive physicians. The importance of accountability and the need to set expectations were consistently cited as a core tenant of remediation. Relatedly, the demonstration of organisational commitment to ensuring a high standard of civility, as well as professional training in such areas as emotional intelligence, self-awareness and teamwork, were also identified as repetitive themes.

While it was generally acknowledged that the recommended strategies for intervention depend on the nature, severity and timing of the transgressions, and that responses should be tailored to each individual situation, a formal approach to the management of disruptive behaviour, coupled with a designated code of conduct, has significant advantages. Since behavioural problems can be symptoms of underlying issues related to drug use, alcoholism, psychiatric conditions and/or burnout, referral for mental health services should be judiciously considered as well.^30^ In this regard, the utility of fitness for duty evaluations, often in conjunction with wellbeing committees, has been well-established in the setting of disruptive behaviour.^29^

The Vanderbilt University experience with the Co-worker Observation Reporting System (CORS), a systems-based platform for reporting of perceived disrespectful and unsafe conduct, demonstrated the importance of peer involvement in the intervention process.^24^ With CORS, selected peers meet with the potentially disrespectful individual, usually in an informal setting, to deliver feedback from a standardised assessment – then allows time for reflection. In their preliminary report, investigators showed that 71% of those individuals who received a report of some behavioural concern were not named in any subsequent reports during a 1-year follow-up period after the behaviour was brought to their attention, and only 3% of the medical staff were associated with a pattern of CORS reports, meaning they received three or more CORS reports within a rolling 3-year period. Their findings clearly emphasise the need for creating an environment of shared purpose and for having organisational support of a zero-tolerance policy for disruptive behaviour.

Swiggart *et al*^25^ subsequently presented prospective data from the same institution on 28 physicians who had been previously cited for unprofessional behaviour and who subsequently attended a Programme for Distressed Physicians (PDP) course, an intensive 3-day programme (followed by three day-long sessions spread over 6 months) which focused on systematic education in emotional intelligence, self-awareness, introspection, interpersonal skills, conflict resolution, leadership, self-care, emotional regulation and mindfulness. Using a specialised 35-question survey – centred on personal demeanour, willingness or ability to keep up with hospital timeliness and tasks, bedside manner and professional behaviour – administered before and after the PDP, the investigators showed that 24 of the 28 physicians demonstrated a significant improvement in their understanding of conduct. This was powerful evidence that unprofessional behaviour in physicians, as observed and reported by their peers and colleagues, can be positively modified by education.

Others have focused on the development of analytical strategies for addressing disruptive behaviours. Williams and Williams proposed the EVLA (Environmentally Valid Learning Approach) model to address the underlying contributing factors and the environment in which unprofessionalism occurs.^26^ Through the use of five fictional cases, they highlighted the complexity in addressing professionalism transgressions and illustrated the need for a broader approach to integrate physician functioning on a physical and psychosocial basis. Similarly, Hastie *et al*^27^ proposed a 3E model that focuses on cultural change through the reinforcement of norms, education and empowerment. What is emphasised is a collective effort from all organisational leaders so that standards are impeccably clear. Likewise, Junga *et al*^28^ developed the four-step ‘DEAL’ model as a practical framework for confronting a disruptive physician, focused on determining the stakes, explaining intent, assessing consequences and leveraging the future. Given the difficult nature of the conversations that commonly arise with those engaged in misconduct, this approach may appeal to those involved in direct on-the-ground management.

The possibility of escalation to more draconian measures including probation, suspension, demotion and/or reporting to the medical board needs to be considered in certain circumstances. Conversations in this setting can be particularly difficult and can be facilitate by ensuring that interventional approaches have been exhausted. While the fear of litigation can lead to hesitation in taking these steps, several court cases have ruled in favour of an organisation’s right to deny or revoke the credentials of a physician because of disruptive behaviour, even if it did not directly cause patient harm.^31^ While it is unestablished which of the aforementioned methods work best, what is clear is that ignoring disruptive behaviour is not an option in the modern healthcare environment.

Studies have repeatedly shown the importance of prevention in limiting disruptive behaviour in the workplace.^32^, ^33^, ^34^ Using qualitative analysis of survey data from nearly 500 physicians and nurses, Lim *et al*^19^ outlined a multipronged three-dimensional approach for tackling disruptive behaviour centred on 1) deterrent measures; 2) development of knowledge and skills including those related to coping, communication, and professionalism; and 3) demonstration of organisational commitment through proper norms, empathising with staff, and structural reforms. By having clearly stated expectations for physician behaviour and policies in place for dealing with problem physicians, organisations have a context from which to address the situation. In this sense, commitment to the highest standards of professionalism must come from all levels of the healthcare system hierarchy.^35^

Lastly, the possibility of mental health disorders and/or substance abuse in contributing to disruptive behaviour must always be considered, as several studies have shown a strong link between the two.^36^^,^^37^ In one study, the prevalence of Axis II cluster B and cluster B diagnosis in disruptive physicians was 50% and 48%, respectively.^37^ Consequently, the use of psychiatric counselling and/or pharmacologic agents might be of utility for some professionals who display a pattern of misconduct.^38^ Future investigations to better understand how potential psychiatric treatment could modify behaviour in this population are warranted so that effective interventional strategies can be optimised in the real-world setting.

The dearth of studies on interventions into disruptive behaviours was demonstrated in this present work. While some individualised approaches were identified that have the potential to be of practical utility, it is worth noting that remediation can take the form of addressing issues within the environment, the leaders and the organisation. The significance of these points is not just to remediate the disruptive physician, but also to identify leadership gaps in the skills and competencies of executive and clinical leaders. The applicability of the strategies identified herein to varying cultural contexts is also unknown and is deserving of further research. For instance, what might be considered disruptive in one culture might not carry the same negative associations in another. Relatedly, it is important to recognise that culturally tailored interventions might be more suitable, depending on the circumstance. Future research on the management of the disruptive physician should thus focus on evaluating interventional strategies in a prospective fashion and considering both internal factors such as those related to mental health and external stressors which might be more systemic in nature.

## Conclusion

Disruptive behaviour will always be an issue in healthcare as long as humans are being human. While some sceptics have sarcastically suggested that management of the disruptive physician is unmanageable in itself and thus a potential exercise in futility, this is not an impossible ordeal.^39^^,^^40^ The strategies that were uncovered in the present study offer a practical means of addressing disruptive behaviour which could aid healthcare leaders in addressing disruption. Yet a notable lack of material exists to counsel, address and support disruptive physicians; and nowhere in healthcare are the stakes arguably higher. Physician misconduct, if left unaddressed, can compromise the quality of patient care and lead to deterioration of morale, which can ultimately subvert the culture of the entire organisation. Although several strategies have been shown to be effective, including those focused on the setting of expectations, organisational commitment and professionalism training, the need to for improvement is obvious. Substantial investment in the development of programmes to address disruptive behaviour and to provide evidence-based remedial support are highly warranted.

## Data availability statement

Data sharing is not applicable to this article as no new data were created or analysed in this study.

## Funding

This work was funded by the University of California, Irvine, M-POWER programme.

## CRediT authorship contribution statement

**Allen M. Chen:** Writing – review & editing, Writing – original draft, Visualization, Validation, Supervision, Software, Resources, Project administration, Methodology, Investigation, Funding acquisition, Formal analysis, Data curation, Conceptualization.

## Declaration of competing interest

The authors declare that they have no known competing financial interests or personal relationships that could have appeared to influence the work reported in this paper.
